# Assessing the antiproliferative effect of biogenic silver chloride nanoparticles on glioblastoma cell lines by quantitative image‐based analysis

**DOI:** 10.1049/nbt2.12038

**Published:** 2021-03-22

**Authors:** Nathalia Müller, Mateus Eugenio, Luciana F. Romão, Jorge Marcondes de Souza, Soniza V. Alves‐Leon, Loraine Campanati, Celso Sant’Anna

**Affiliations:** ^1^ Laboratory of Microscopy Applied to Life Science ‐ Lamav National Institute of Metrology Quality and Technology Duque de Caxias RJ Brazil; ^2^ Laboratory of Cellular Morphogenesis Federal University of Rio de Janeiro Rio de Janeiro RJ Brazil; ^3^ University Hospital Clementino Fraga Filho Federal University of Rio de Janeiro Rio de Janeiro RJ Brazil

## Abstract

Glioblastoma is the most life‐threatening tumour of the central nervous system. Temozolomide (TMZ) is the first‐choice oral drug for the treatment of glioblastoma, although it shows low efficacy. Silver nanoparticles (AgNPs) have been shown to exhibit biocidal activity in a variety of microorganisms, including some pathogenic microorganisms. Herein, the antiproliferative effect of AgCl‐NPs on glioblastoma cell lines (GBM02 and GBM11) and on astrocytes was evaluated through automated quantitative image‐based analysis (HCA) of the cells. The cells were treated with 0.1‐5.0 μg/ml AgCl‐NPs or with 9.7‐48.5 μg/ml TMZ. Cells that received combined treatment were also analysed. At a maximum tested concentration of AgCl‐NPs, GBM02 and GBM11, the growth decreased by 93% and 40%, respectively, following 72 h of treatment. TMZ treatment decreased the proliferation of GBM02 and GBM11 cells by 58% and 34%, respectively. Combinations of AgCl‐NPs and TMZ showed intermediate antiproliferative effects; the lowest concentrations caused an inhibition similar to that obtained with TMZ, and the highest concentrations caused inhibition similar to that obtained with AgCl‐NPs alone. No significant changes in astrocyte proliferation were observed. The authors’ findings showed that HCA is a fast and reliable approach that can be used to evaluate the antiproliferative effect of the nanoparticles at the single‐cell level and that AgCl‐NPs are promising agents for glioblastoma treatment.

## 1 Introduction

Glioblastoma (GBM), which is classified as a grade IV astrocytoma by the World Health Organization (WHO), is a fatal malignant brain tumour that occurs in adults. Malignant tumours of the brain and central nervous system account for fewer than 3% of new cases of cancer worldwide, but the incidence rate of such tumours is similar to the mortality rate [1]. Treatment might involve surgery, radiotherapy, immunotherapy and chemotherapy. However, considering the tendency of such tumours to infiltrate the brain tissue, therapy is difficult and only prolongs the patient survival by approximately 12 months [2].

Temozolomide (TMZ) is a lipophilic chemotherapeutic agent that traverses the blood‐brain barrier and is used as a first‐line treatment in glioblastoma cases [2]. Generally, this drug is administered together with radiotherapy or as a stand‐alone therapy [3]. In plasma, TMZ, which is a prodrug, is quickly transformed into a derivative of imidazotetrazinone and into the methyldiazonium ion, a DNA alkylating agent that interrupts the cell cycle [2]. As a consequence of treatment, approximately 90% of patients develop resistance to TMZ, and tumour recurrence is inevitable [4].

Nanomedicine is a promising area of biotechnology in which nanotechnology is applied to medical equipment and treatments. Metal nanoparticles (MNPs) bring new expectations for cancer treatment because they improve treatment by acting as a drug delivery system and, in diagnosis, as a biosensor [5]. Nanoparticles (NPs) have been used in various fields of medicine because of their unique properties. MNPs of gold, silver, titanium and zinc attract attention due to their size, which favours a high surface area, and their unique physical and chemical properties ensure interaction with cell membranes [5, 6].

MNPs, including metallic silver nanoparticles (AgNPs) and silver chloride nanoparticles (AgCl‐NPs), are known for their great potential as antimicrobial agents against bacteria, fungi and some protozoa and their low in vivo toxicity to mammalian cells [7, 8]. In cancer biology, silver‐based nanoparticles are increasingly drawing attention because of their already described cytotoxic effects on various tumour cells, including human breast cancer cells, Dalton’s lymphoma and U87 MG glioblastoma cells [9, 10]. The observed effects of silver‐based nanoparticles include induction of DNA damage, oxidative stress and apoptosis. The antitumour effect of AgNPs has been measured *using* in vivo and in vitro assays; however, to date, there are no reports of the efficacy of AgCl‐NPs in tumour cells [11, 12].

MNPs are produced by physical and chemical synthesis. The former requires special equipments to maintain high temperature and pressure for production, and the latter uses chemical agents that are harmful to the environment [13, 14]. In contrast, biological synthesis―‘green synthesis’―is an environmentally friendly and economical process that does not require the use of hazardous chemicals, as the cellular metabolism itself is responsible for the reduction of metal ions into nanoparticles. The biological production of MNPs from a wide variety of microorganisms and plant extracts has been reported [13]. These aspects of biosynthesis make it a great methodological option for biomedical applications.

A large number of microorganisms and plant species are capable of reducing metal salts into nanoparticles [14], and bacterial biosynthesis has a good cost‐benefit ratio compared to that of other techniques used in biotechnology. In general, the biosynthesis eliminates the use of toxic substances since the bacteria possess all the enzymes needed for the process, and there are well‐established bacterial culture techniques. Bacteria are known to efficiently absorb metals and magnetic particles from the environment. Another great advantage of this type of synthesis is its high yield, since multiple new generations of bacteria are produced within a few hours [15].

Similar to TMZ, chemically produced AgNPs have been reported to be genotoxic to fibroblasts and to GBM U251 cells in vitro [16]. The cytotoxic potential of biological nanoparticles has been evaluated in recent years, and its effects on pathogens and tumour cells have been demonstrated [8].

*Bacillus* is the most abundant genus in the rhizosphere, and each species in this genus produces enzymes that act via hydrolysis, oxidation or reduction. *B. vi* has been used industrially for over 50 years since it has a high capacity for exoenzyme production. The production of silver‐based nanoparticles by members of the genus *Bacillus* has been described. [17] e [18] reported the production of AgNPs using *Bacillus megaterium*, and our group demonstrated the production of AgCl‐NPs by the same bacteria [17, 18, 19].

In this work, we used two GMB cell lines and human astrocytes to evaluate the effects of AgCl‐NPs derived from *B. megaterium*. The cells were treated with a range of concentrations of AgCl‐NPs and TMZ and with combinations of the two agents. Cell proliferation was monitored using an automated HCA assay, a rapid and accurate method for morphological and quantitative studies [20].

## 2 MATERIALS AND METHODS

### 2.1 Bacterial culture, biosynthesis and isolation of AgCl‐NPs

*B. megaterium* ATCC 14,581 was grown in modified nutrient broth (1% peptone, 0.5% meat extract and 0.5% yeast extract) for 24 h. The production of AgCl‐NPs was conducted as reported previously by our group [19]. Briefly, the culture was maintained in the presence of 3.5 mM silver nitrate (AgNO_3_) for 7 days at 37°C with agitation at 150 rpm in the absence of light. Following the confirmation of AgCl‐NP synthesis by a change in the colour of the medium, the cells were removed from the medium by centrifugation at 2720 x g for 10 min at room temperature, and AgCl‐NPs were isolated from the supernatant by centrifugation at 38,360 x g for 20 min at room temperature. The pellet containing NPs was resuspended and washed with 2% sodium citrate solution, pH 8.0. This step was repeated until the supernatant appeared clear.

### 2.2 Culture of Glioblastoma Cells and Astrocytes

GBM02 and GBM11 cells were isolated from surgical specimens obtained from patients who were referred for surgical tumour removal. These lines were established in the Laboratory of Cell Morphogenesis [21] with the patients consent and with the approval of the Ethics Committee of the Brazilian Ministry of Health and the Ethics Committee of Clementino Fraga Filho Hospital (CEP‐HUCFF n. 002/01). Primary human astrocytes, which are non‐tumor cells, were isolated from tissue resected from patients who underwent surgical treatment of the temporal lobe epilepsy associated with hippocampal sclerosis. The cells were obtained with patient consent to their use in the isolation of astrocytes, and the procedures were approved by the Ethics Committee of the Brazilian Ministry of Health under CRI ‐ CEP/HUCFF n. 060/05.

GBM02 and GBM11 cells were cultivated in DMEM‐F12 medium (supplemented with 10% foetal bovine serum (FBS) and maintained at 37°C (5% CO_2_) until they had reached 80% confluence. The cells were then detached from the flasks by incubation in a solution of 0.05% trypsin/0.02% ethylenediaminetetraacetic acid (EDTA) for 5 min at 37°C.

All patients gave written consent for participation in the study, and the procedures were conducted in accordance with the guidelines set forth by the Brazilian Ministry of Health Ethics Committee (CONEP 2340). Cultures of astrocytes were established from healthy cortical brain tissue as described previously [22]. Briefly, tissue samples were washed in DMEM‐F12, mechanically dissociated, cut into small pieces with a sterile scalpel, and incubated in 10 ml of 0.25% trypsin (in water) at 37°C for 10 min. After centrifugation, the cell pellet was resuspended in DMEM‐F12 supplemented with 10% FBS, and the cells were plated in tissue culture flasks and allowed to adhere at 37°C (5% CO_2_) for 2 h. Non‐adherent astrocytes were then transferred to fresh culture flasks previously coated with 0.1% poly‐L‐lysine. When the cultures reached 80% confluence, astrocytes were detached using 0.05% trypsin/0.02% EDTA.

### 2.3 Antiproliferation Assay by HCA

For cell proliferation analysis, GBM02 and GBM11 cell lines and primary astrocytes were plated in black, flat‐bottom 96‐well plates at a density of 10^3^ cells/well in 300 μL/well DMEM/F12 supplemented with 10% FBS containing 0.5 μg/ml Hoechst 33,258 dye and incubated at 37°C. Twenty‐four hours after plating, the cells were incubated with various concentrations of AgCl‐NPs (0.1, 0.5, 1.0, 2.5 and 5.0 μg/ml), TMZ (9.7, 19.4, 29.1, 38.5 and 48.5 μg/ml) or with combinations of AgCl‐NPs and TMZ (0.1 + 9.7, 0.5 + 19.4, 1.0 + 29.1, 2.5 + 38.5 and 5.0 + 48.5 μg/ml) for 24, 48 and 72 h at 37°C (5% CO_2_). The maximum concentration of TMZ used in the experiments was compatible with that recommended by the Guidelines for Clinical Practice in Oncology for the treatment of central nervous system tumours [23]. An untreated control was performed in the absence of nanoparticles or TMZ. Images were obtained using an IN Cell Analyser 2000 system at 0, 24, 48 and 72 h after treatment of the control and treated cells. The images were acquired in six random fields within each well using the DAPI filter (spectral region between 410 and 480 nm) with a 10× objective.

### 2.4 Analysis of Cell Proliferation by HCA

Images were acquired as described above. After segmentation, the objects (nuclei) were automatically quantified to estimate cell proliferation. The number of stained nuclei was counted using IN Cell Investigation software. Cell proliferation after 24, 48 and 72 h of treatment was calculated as a percentage relative to the number of cell nuclei counted in the control condition (untreated cells). Inhibitory potency was also assessed by determining the half‐maximal inhibitory concentration (IC50).

### 2.5 Statistical Analysis

Assays of the effects of the tested agents on cell proliferation were performed in triplicate, and the results are expressed as the mean ± standard deviation. Data were analysed in GraphPad Prism seven by one‐way ANOVA followed by Tukey's test, considering statistical significance at *p* ≤ 0.05.

## 3 RESULTS

### 3.1 Proliferation of Tumour and Non‐tumor Cells After Exposure to AgCl‐NPs, TMZ and AgCl‐NPs + TMZ

**FIGURE 1:**
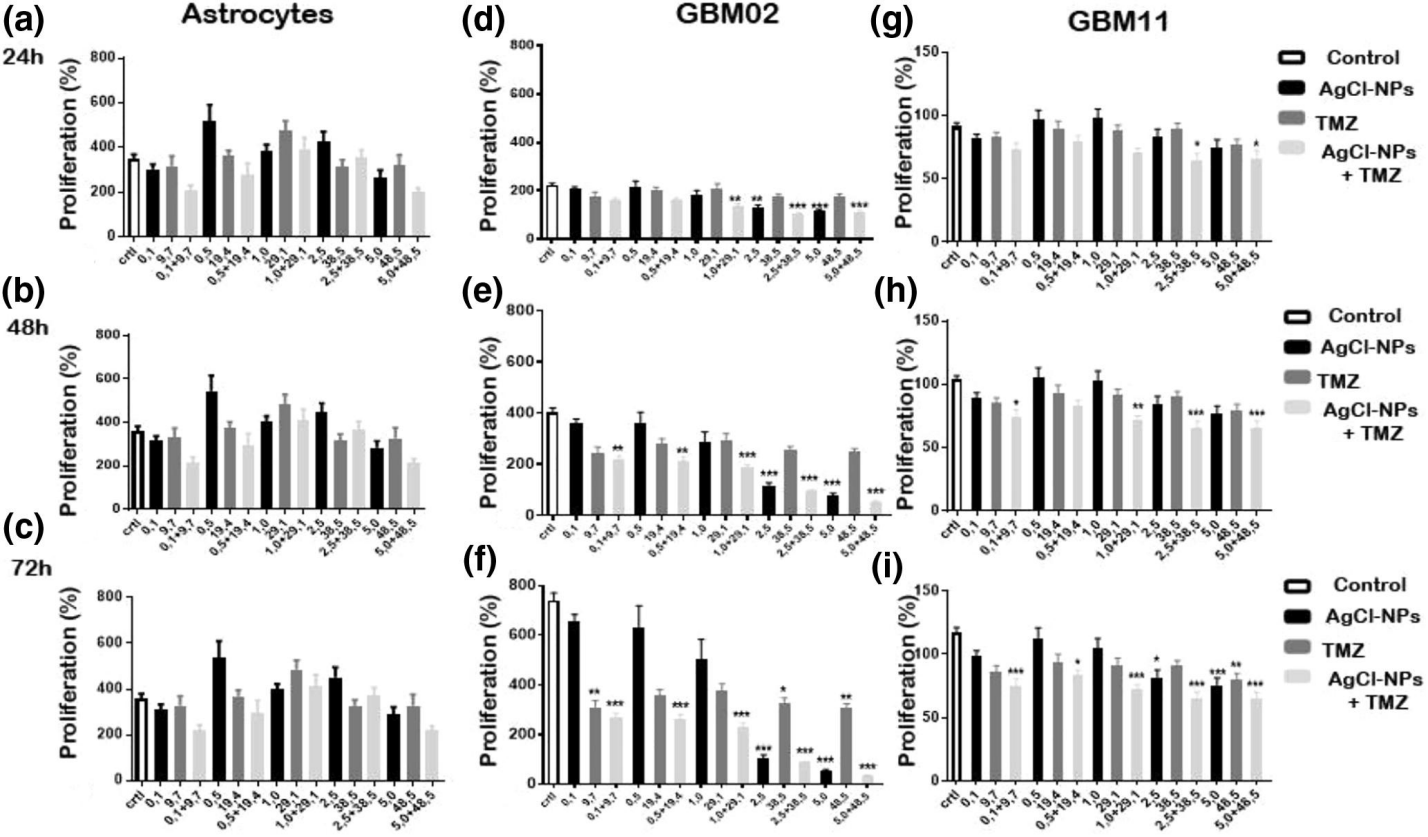
Antiproliferation assay. GBM02 and GBM11 cells and primary astrocytes were treated with AgCl‐NPs, TMZ or a combination of both at concentrations ranging from 0.1 to 5.0 μg/ml (AgCl‐NPs) and 9.7 to 48.5 μg/ml (TMZ) for 24, 48 and 72 h. Cell proliferation was calculated as a percentage of the number of cells (nuclei) counted under control (untreated) conditions. Groups with significant differences relative to the control group are indicated by * for p < 0.05, ** for p < 0.01 and *** for p < 0.001

AgCl‐NPs produced by *B. megaterium* were evaluated for their effect on the proliferation of human astrocytes and GBM02 and GBM11 cells. Thte results are presented as the per cent inhibition of proliferation (Figure 1 and Supplementary S1). Per cent inhibition was obtained from the numerical ratio of the nuclei counted for the same cell type on day 0 (control).

Twenty‐four hours after treatment, AgCl‐NPs at 1.0 μg/ml caused a reduction of 11.8 (±39.1)% in the growth of GBM02 cells and a reduction of 45 (±21)% at 5.0 μg/ml. After 48 h of treatment with AgCl‐NPs at 2.5 μg/ml, the proliferation of GBM02 cells was reduced by 71 (±9.8)%. Treatment with TMZ for 24 h at the maximum concentration (48.5 μg/ml) used in glioblastoma treatment resulted in a reduction of 15 (±26.3)%.

After 72 h, treatment with 5.0 μg/ml AgCl‐NPs alone or in combination with TMZ (5.0 + 48.5 μg/ml) caused growth inhibition of more than 93%, whereas 48.5 μg/ml TMZ alone reduced the proliferation of GBM02 cells by 58.3%. Treatment with 9.7, 38.5 and 48.5 μg/ml TMZ for 72 h resulted in antiproliferative effects ranging from 56% to 58% relative to the control. Combined treatment with 1.0 + 29.1 μg/ml, 2.5 + 38.5 μg/ml and 5.0 + 48.5 μg/ml AgCl‐NPs + TMZ resulted in significant reductions in the proliferation of GBM02 cells relative to the control at all times; treatment with 2.5 or 5.0 μg/ml AgCl‐NPs alone also resulted in a significant reduction in proliferation relative to the control during the course of the experiment.

For GBM11 cells, 72 h of treatment with the maximum tested concentration of TMZ led to a 34.6 (±20.1)% reduction in proliferation, whereas AgCl‐NP treatment led to a reduction of 40.5 (±20)%; the difference in the effects of the two treatments was not significant. Combined treatment (72 h) at the maximum tested concentrations of the two agents (5.0 + 48.5 μg/ml AgCl‐NPs + TMZ, respectively) led to a significant reduction of 47.8% relative to the control.

After 72 h, combined treatment at all tested concentrations produced a significant inhibition of proliferation relative to control cells (the resulting percent inhibition, in ascending order of concentration of the applied agents, was 40.6%, 33.1%, 42.6%, 47.9% and 47.8%). Treatment with AgCl‐NPs alone had a statistically significant effect on proliferation compared with the control cells only at concentrations of 2.5 μg/ml (34%) and 5.0 μg/ml (40.5%) after 72 h. Combined treatment with 2.5 + 38.5 μg/ml and 5.0 ± 48.5 μg/ml AgCl‐NPs + TMZ significantly reduced tumour cell proliferation by 33.5% and 31.8%, respectively, after 24 h.

Astrocytes were used as a model of healthy cells to test the selectivity of the treatments. Neither TMZ, AgCl‐NPs or AgCl‐NPs + TMZ negatively affected the proliferation of astrocytes compared with control cells.

### 3.2 Half‐maximal Inhibitory Concentrations of AgCl‐NPs, TMZ and AgCl‐NPs + TMZ for the Proliferation of GBM Cell Lines and Astrocytes

To compare the potency of AgCl‐NPs and TMZ treatments on the growth of GBM cell lines and astrocytes, we determined the IC_50_ of the compounds (Table 1). Higher concentrations of TMZ and AgCl‐NPs were required to inhibit the growth of GBM11 cells than to inhibit the growth of GBM02 cells at all time points with the exception of 72 h where the same apparent IC_50_ values (1.4 μg/ml) were observed for both cell lines. For both GBM cell lines, the IC50 of AgCl‐NPs was at least 3 times lower than that of TMZ.

**TABLE 1:** IC_50_ (μg/ml) according to treatment

Treatment	Time (h)	IC_50_ GBM02	IC_50_ GBM11	IC_50_ astrocytes
AgCl‐NPs	24	4.0	5.5	154.7
AgCl‐NPs	48	1.8	5.4	181.6
AgCl‐NPs	72	1.4	4.4	50.6
TMZ	24	14.3	21.4	23.4
TMZ	48	11.8	27.8	21.2
TMZ	72	1.4	28.7	20.9
AgCl‐NPs + TMZ	24	4.7	5.4	28.2
AgCl‐NPs + TMZ	48	1.3	6.5	23.2
AgCl‐NPs + TMZ	72	ND	5.7	29.4

The concentration of AgCl‐NPs required to reduce the number of astrocytes by 50% was at least twofold higher than the TMZ concentration, showing that the treatment with AgCl‐NPs had less deleterious effects on non‐tumor cells than did the treatment with TMZ. No significant difference between combined treatment (AgCl‐NPs + TMZ) and treatment with TMZ alone was found for the GBM cells at any of the time points tested. In astrocytes, exposure to AgCl‐NPs + TMZ under equivalent conditions resulted in an increase in the inhibitory activity.

## 4 DISCUSSION

The biosynthesis of AgCl‐NPs used in this work and their characterisation by spectroscopic and electron microscopy approaches were previously reported by our group [19]. In summary, AgCl‐NPs were produced from Gram‐positive bacterium *Bacillus megaterium* cultures. The AgCl‐NPs bioproduction was firstly evidenced by a medium colour change (from pale yellow to dark brown), and then, was further confirmed by UV‐VIS analyses. Absorbance in the spectrum ranging from 380 to 450 nm, with maximum absorbance at 416 nm, was determined. Energy dispersive X‐ray (EDS) spectroscopy analyses of nanoparticles showed peaks at 3  and 2.75 keV, corresponding to silver and chlorine atoms, respectively. X‐ray diffraction (XRD) analysis accurately promoted the chemical identification of AgCl‐NPs. The obtained diffraction profile was consistent with the JCPDS standards (JCPDS: 31‐1238) for AgCl–NPs. Morphometric analyses by transmission electron microscopy (TEM) showed an average diameter of 13 ± 4 nm. The shape factor (circularity) showed that approximately 80% of nanoparticles had a spherical morphology.

The rate of proliferation of the tumour and non‐tumor cells treated with AgCl‐NPs was determined by image‐based quantitative cellular analysis (HCA) in which the counting of the nuclei stained with Hoechst 33,258 was performed automatically using digital fluorescent images. The AgCl‐NPs biosynthesis―from *B. megaterium* and their characterisation by spectroscopy and electron microscopy―was previously reported by our group [19]. Automated image‐based analysis has been used in the field of cancer biology for years, and its main area of application has been drug discovery. The HCA method was used in this study instead of the traditional methods to increase the reliability of the analysis of many samples and reduce time and minimise operator bias [20].

Comparison of cell growth showed that the treatments used in this study had a greater impact on tumour cells than astrocytes. The tested cell lines responded differentially, with the tested agents showing more efficiency in GBM02 cells than in GBM11 cells. Recently, our group evaluated the antiproliferative effect of silver/silver chloride nanoparticles (Ag/AgCl‐NPs) isolated from fungi on GBM02 cells and astrocytes [10]. The maximum tested concentrations of these agents, which were 5.0 μg/ml Ag/AgCl‐NPs and 48.5 μg/ml TMZ, inhibited the proliferation of GBM02 cells by 82% and 62%, respectively. In the evaluation of the impact of the treatment on astrocytes, the inhibition of astrocyte growth was found to be 27% (3‐fold lower than for tumour cells), while the highest tested concentration of TMZ inhibited the proliferation of astrocytes by 37%.

In an *in ovo* assay, [24] showed a reduction of 35% in GBM U87 proliferation using 40 μg/ml AgNPs, a concentration of AgCl‐NPs is eight times higher than that used in the present study and with lower inhibition results. It is important to emphasize that untreated GBM02 cells showed higher proliferation than did untreated GBM11 cells [24]. Figure 1 shows that while the GBM02 population increased fourfold during a period of 72 h in the current study, the GBM11 population increased by only slightly more than 10%. Despite its high replication rate, we observed that GBM02 was more responsive to the three treatments. This difference may be due to intrinsic genetic diversity, a characteristic of glioblastoma. Analysis of the mRNA expression has demonstrated the existence of four GBM subtypes (classical, proneural, neural and mesenchymal), each with distinct patterns of disease progression, survival and response to therapy [25]. In astrocytes, none of the concentrations tested under the three treatment conditions caused a significant reduction in cell proliferation relative to the control (Figure 1). Only at concentrations of AgCl‐NPs and TMZ of 0.1 + 9.7 μg/ml and 5.0 + 48.5 μg/ml concentrations of AgCl‐NPs and TMZ during the three studied times did we observe a non‐significant reduction in cell proliferation; at all other concentrations, astrocyte proliferation was not influenced (Figure 1). It was noticeable that after 72 h, the results obtained using AgCl‐NPs to treat both glioblastoma cell lines were better than those observed for the cell lines treated with TMZ alone. Our results show that TMZ and AgCl‐NPs did not negatively affect astrocyte proliferation, suggesting that AgCl‐NPs may be act as good candidates for more specific assays such as assays of viability and evaluation of morphological changes.

In general, research on the use of NPs in the field of oncology is associated with the development and improvement of diagnostic methods with the role of NPs in treatment as ‘*drug delivery*’ systems and ‘target nanosystems’; however, assays that evaluated various uncoated NPs, including MNPs, have shown promising results [26]. Combined therapy may be a promising strategy for improving therapeutic efficacy and eliminating undesirable side effects. The use of combinations of different drugs provides several advantages, such as synergistic drug effects and reversal of drug resistance. [27] evaluated the effect of combination therapy with camptothecin (CPT), a topoisomerase inhibitor used in the treatment of various tumours, and AgNPs in vivo. The combination of CPT and AgNPs significantly inhibited cell proliferation and increased cytotoxicity and apoptosis through the generation of reactive oxygen species (ROS), the release of dehydrogenated lactate, alteration of the mitochondrial membrane potential and activation of caspases 9, 6 and 3. In addition, the authors observed an increase in the level of pro‐oxidants and a reduction in the levels of antioxidants [27]. Recently, AgNPs in combination with salinomycin were found to induce increased apoptotic and autophagic activity in ovarian cancer cells. The combination of gemcitabine and AgNPs also increased apoptotic potential, oxidative stress, and mitochondrial membrane potential loss, increased pro‐apoptotic expression, and promoted negative regulation of anti‐apoptotic genes in ovarian cancer cells [27, 28].

Our results suggest that the combined use of AgCl‐NPs and TMZ may be a viable approach to GBM treatment. Such a combination had a cytotoxic effect superior to that of TMZ alone and the results of the combination treatment were closer to those obtained with AgCl‐NPs alone. Studies aimed at better understanding how each of these agents acts mechanistically on cellular metabolism are necessary.

Recently, our group reported the cytotoxic potential of AgCl‐NPs in three‐dimensional culture spheroids of human adipose tissue stem cells (ASCs). The effects of sublethal doses (5, 10 and 25 μg/ml) of AgCl‐NPs were analysed for 21 days. Light and electron microscopy showed no change in spheroid composition, cell morphology or viability at any dose studied. However, between days 1 and 7 of AgCl‐NP treatment, increased ROS levels, as well as changes in the secretory profiles of interleukins (pro‐inflammatory interleukins IL‐6, IL‐1b and IL‐8 and anti‐inflammatory interleukin IL‐10) and TGF‐β, were observed in the supernatants of the ASC spheroids. The possibility of modulation of the microenvironment and remodelling of the extracellular matrix suggests the need for further studies [19].

Further assays are needed to determine whether the effect of AgCl‐NPs is cytostatic, cytotoxic or both. In addition, morphological and ultrastructural analyses will help identify possible structural changes and likely targets of AgCl‐NPs in the intracellular context.

## 5 CONCLUSION

In conclusion, the results of the present study show that AgCl‐NPs produced by *B. megaterium* are promising antitumour agents that are capable of altering the proliferation of glioblastoma cells with a minimal effect on astrocytes.

## Supporting information

Supplementary Material
