# Supplementary material for: Assessing the antiproliferative effect of biogenic silver chloride nanoparticles on glioblastoma cell lines by quantitative image‐based analysis
Source: IET Nanobiotechnol. 2021 Mar 22;15(6):558–64. doi: 10.1049/nbt2.12038 (PMC8675776; doi:10.1049/nbt2.12038)
Supplement: Supplementary file 1 — Supplementary Material [file NBT2-15-558-s001.docx]

| Treatment | Astrocytes | | | GBM02 | | | GBM11 | | |
| --- | --- | --- | --- | --- | --- | --- | --- | --- | --- |
| mg/ml | **24 h** | **48 h** | **72 h** | **24 h** | **48 h** | **72 h** | **24 h** | **48 h** | **72 h** |
| 0.1 | -18.4 (±57.0) | -19.0 (±58.5) | -17.0 (±54.3) | 0.91 (±28.5) | 5.7 (±29.6) | 11.1 (26,1) | 14.2 (±24.7) | 16.1 (±24.0) | 19.5 (±21.3) |
| 9.7 | -24.2 (±79.7) | -25.4 (±76.8) | -23.5 (±74.9) | 13.4 (±42.5) | 34.9 (±30.6) | 56.4 (±20.7) | 12.5 (±28.3) | 19.4 (±25.5) | 28.9 (±21.3) |
| 0.1+9.7 | 21.8 (±30.7) | 20.4 (±31.7) | 19.5 (±32.0) | 22.0 (±29.9) | 41.6 (±22.9) | 62.5 (±14.9) | 26.4 (±16.1) | 32.5 (±18.3) | 40.6 (±15.9) |
| 0.5 | -103.7 (±117.4) | -104.6 (±110.0) | -102.2 (±108.9) | -6.7 (±50.8) | 3.0 (±59.0) | 11.9 (±54.7) | -1.3 (±39.2) | 2.4 (±35.8) | 7.8 (±32.7) |
| 19.4 | -44.3 (±65.5) | -46.36 (±67.6) | -42.17 (±62.22) | 2.9 (±44.7) | 25.0 (±37.3) | 50.4 (±22.03) | 8.4 (±19.3) | 14.4 (±20.6) | 25.0 (±16.75) |
| 0.5+19.4 | -8.0 (±74.0) | -12.5 (74.4) | -12.4 (±69.3) | 22.0 (±30.7) | 43.6 (±19.5) | 64.1 (±11.7) | 18.5 (±17.2) | 23.7 (±18.8) | 33.1 (±14.8) |
| 1.0 | -51.8 (±71.7) | -54.8 (±70.2) | -51.8 (±70.4) | 11.8 (±39.1) | 26.5 (±36.9) | 32.9 (±38.4) | -4.1 (±45.3) | 1.9 (±43.9) | 13.3 (±34.7) |
| 29.1 | -101.9 (±127.1) | -100.8 (±127.6) | -99.3 (±126.3) | -3.9 (±52.7) | 19.2 (±46.9) | 47.0 (±27.8) | 8.8 (±29.2) | 15.2 (±26.7) | 25.6 (±23.7) |
| 1.0+29.1 | -57.9 (±120.3) | -57.9 (123.7) | -56.15 (±112.9) | 35.1 (±22.8) | 51.2 (17.8) | 68.7 (11.12) | 28.7 (±9.0) | 35.0 (±9.9) | 42.6 (±8.3) |
| 2.5 | -66.9 (±88.9) | -70.6 (±91.1) | -70.8 (±89.7) | 38.6 (±18.3) | 71.0 (±9.8) | 86.5 (±5.4) | 14.7 (±28.3) | 22.3 (±24.5) | 34.0 (±20.0) |
| 38.5 | -26.5 (±79.6) | -23.9 (±76.2) | -25.4 (±77.8) | 17.8 (±20.11) | 34.9 (±16.4) | 56.4 (12.2) | 7.87 (25.6) | 16.5 (±23.7) | 26.0 (±19.5) |
| 2.5+38.5 | -50.2 (±103.1) | -49.7 (±104.3) | -48.5 (±99.6) | 49.9 (±21.9) | 74.4 (±9.1) | 88.1 (±2.8) | 33.5 (±20.4) | 39.6 (±17.5) | 47.9 (±13.4) |
| 5.0 | -3.3 (±48.5) | -6.7 (45.1) | -8.64 (±48.33) | 45.0 (±21.0) | 79.8 (7.4) | 93.2 (±2.9) | 24.0 (±26.7) | 30.5 (±22.2) | 40.5 (±20.0) |
| 48.5 | -31.5 (±102.3) | -31.72 (±103.8) | -32.87 (±104.5) | 15.0 (±26.3) | 34.4 (±22.3) | 58.3 (±11.9) | 20.2 (±23.0) | 25.6 (±23.4) | 34.6 (±20.1) |
| 5.0+48.5 | 22.4 (±39.9) | 21.0 (±40.1) | 20.15 (±34.5) | 47.0 (±24.9) | 86.3 (±6.6) | 95.5 (±2.3) | 31.8 (±28.0) | 39.0 (±22.7) | 47.8 (±13.4) |

Table S1 – Percent inhibition of the growth of tumor cells and astrocytes after treatment with TMZ and AgCl-NPs. “ – ” indicates the conditions under which no growth inhibition was observed.
